# Clinical Phase I/II Study: Local Disease Control and Survival in Locally Advanced Pancreatic Cancer Treated with Electrochemotherapy

**DOI:** 10.3390/jcm10061305

**Published:** 2021-03-22

**Authors:** Francesco Izzo, Vincenza Granata, Roberta Fusco, Valeria D'Alessio, Antonella Petrillo, Secondo Lastoria, Mauro Piccirillo, Vittorio Albino, Andrea Belli, Salvatore Tafuto, Antonio Avallone, Renato Patrone, Raffaele Palaia

**Affiliations:** 1 Hepatobiliary Surgical Oncology Unit, “Istituto Nazionale Tumori IRCCS Fondazione Pascale-IRCCS di Napoli”, 80131 Naples, Italy; f.izzo@istitutotumori.na.it (F.I.); m.piccirillo@istitutotumori.na.it (M.P.); v.albino@istitutotumori.na.it (V.A.); a.belli@istitutotumori.na.it (A.B.); r.palaia@istitutotumori.na.it (R.P.); 2 Radiodiodiagnostic Unit, “Istituto Nazionale Tumori IRCCS Fondazione Pascale-IRCCS di Napoli”, 80131 Naples, Italy; v.granata@istitutotumori.na.it (V.G.); a.petrillo@istitutotumori.na.it (A.P.); 3 Research and Development Division, Igea SpA, 41012 Carpi, Italy; v.dalessio@igeamedical.com; 4 Nuclear Medicine Unit, “Istituto Nazionale Tumori IRCCS Fondazione Pascale-IRCCS di Napoli”, 80131 Naples, Italy; s.lastoria@istitutotumori.na.it; 5 Sarcomas and Rare Tumors Unit, “Istituto Nazionale Tumori IRCCS Fondazione Pascale-IRCCS di Napoli”, 80131 Naples, Italy; s.tafuto@istututotumori.na.it; 6 Abdominal Oncology Unit, “Istituto Nazionale Tumori IRCCS Fondazione Pascale-IRCCS di Napoli”, 80131 Naples, Italy; a.avallone@istitutotumori.na.it; 7 PhD ICHT, University of Naples Federico II, 80131 Naples, Italy; dott.patrone@gmail.com

**Keywords:** pancreatic cancer, reversible electroporation, response assessment, variable geometry, planning

## Abstract

Objective. To assess local disease control rates (LDCR) and overall survival (OS) in locally advanced pancreatic cancer (LAPC) treated with electrochemotherapy (ECT). Methods. Electrochemotherapy with bleomycin was performed in 25 LAPC patients who underwent baseline Magnetic Resonance Imaging (MRI) and/or Computed Tomography (CT) and Position Emission Tomography (PET) scans before ECT and 1 and 6 months post ECT. LDCR were assessed using Response Evaluation Criteria in Solid Tumors (RECIST 1.1) and Choi criteria. Needle electrodes with fixed linear (N-30-4B) or fixed hexagonal configurations (N-30-HG or I-40-HG or H-30-ST) or variable geometry (VGD1230 or VGD1240) (IGEA S.p.A., Carpi, Italy) were used to apply electric pulses. Pain evaluation was performed pre-ECT, after 1 month and after 6 months with ECT. Overall survival estimates were calculated by means of a Kaplan-Meier analysis. Results. At 1 month after ECT, 76% of patients were in partial response (PR) and 20% in stable disease (SD). Six months after ECT, 44.0% patients were still in PR and 12.0% in SD. A LDCR of 56.0% was reached six months after ECT: 13 patients treated with fixed geometry had a LDCR of 46.1%, while for the 12 patients treated with variable geometry, the LDCR was 66.7%. The overall survival median value was 11.5 months: for patients treated with fixed geometry the OS was 6 months, while for patients treated with variable geometry it was 12 months. Electrochemotherapy was well-tolerated and abdominal pain was rapidly resolved. Conclusions. Electrochemotherapy obtained good results in terms of LDCR and OS in LAPC. Multiple needle insertion in a variable geometry configuration optimized by pre-treatment planning determined an increase in LDCR and OS compared to a fixed geometry configuration.

## 1. Introduction

Adenocarcinoma, which originates in the ducts that carry digestive enzymes, is the most common and aggressive type of pancreatic cancers. Surgical resection is a potentially curative option but unfortunately, over 80% of patients have unresectable, locally advanced or metastatic pancreatic cancer [1]. The locally advanced pancreatic cancer (LAPC) is defined as non-metastasized but unresectable disease due to involvement of the coeliac trunk or superior mesenteric artery (stage III disease) [2]. Radiotherapy and chemotherapy such as gemcitabine alone or in combination with other chemotherapy agents are the standard therapy [1,2,3,4,5]. Because only a limited group of patients responds to chemotherapy, other therapies to be administrated after the end of chemotherapy were explored to obtain tumor debulking or interstitial ablation [6,7] such as irreversible [8,9,10,11] and reversible electroporation [12,13,14,15,16,17,18]. Reversible electroporation, known as electrochemotherapy (ECT), has been used to increase uptake into tumor cells of low doses of non-permeant or poorly permeant chemotherapeutic drugs [18]. Electrochemotherapy with bleomycin has been shown to be very effective in different cutaneous and subcutaneous tumors such as melanoma and chest wall breast cancer recurrence or for the treatment of squamous cell carcinoma of the head and neck when compared with bleomycin therapy alone [19].

Electrochemotherapy effectiveness on pancreatic cancer was already demonstrated in preclinical studies [16,20], and in our previous studies, safety and effectiveness of ECT in patients with locally advanced pancreatic cancer were investigated [21,22]. The feasibility of percutaneous electrochemotherapy in the treatment of portal vein tumor thrombosis at the hepatic hilum in patients with hepatocellular carcinoma with cirrhosis was already investigated [23,24].

The primary endpoint of the study was to evaluate local disease control and overall survival at 24 months in patients with LAPC with stable disease or partial response after chemotherapy, not eligible for surgery, and then treated with ECT. The feasibility and safety of ECT have been also evaluated as secondary endpoints.

## 2. Materials and Methods

### 2.1.Study Population

In this prospective clinical phase I/II study, 25 patients (13 female and 12 male, median age 68.5 ± 8.5 years) with a diagnosis of locally advanced pancreatic adenocarcinoma were enrolled from November 2011 to December 2019. The study was approved by the local ethical committee (research registry 60) and all patients enrolled have signed the informed consent. All patients were discussed by the local multidisciplinary team, and considering the locally advanced nature of the disease, no patient was suitable for surgery; for this reason, all patients underwent neoadjuvant systemic chemotherapy and after were re-staged with imaging. All patients enrolled received systemic chemotherapy before ECT treatment. The patients with stable disease or partial response after chemotherapy who were not eligible for surgery as confirmed by clinical and radiological examination were considered candidates for ECT treatment.

Two chemotherapy regimens were adopted for systemic chemotherapy before ECT treatment: gemcitabine + oxaliplatin (GEMOX) or 5-FU/leucovorin, irinotecan, and oxaliplatin (FOLFIRINOX). Details of chemotherapy regimens were reported in our previous publication [20]. Seventeen (17/25, 68.0%) patients were subjected to GEMOX, and eight patients (8/25, 32.0%) were treated with FOLFIRINOX before ECT treatment (median time between the start of chemotherapy and ECT was 121 days, range 112–140). Patients in progressive disease after ECT underwent conventional systemic treatment (FOLFIRINOX or GEMOX).

Inclusion criteria were: 18–80 years, favorable mental health, life expectancy at least of 3 months, pancreatic adenocarcinoma with histological confirmation, locally advanced disease (stage III) confirmed by radiological assessment, not eligible for curative surgery. Exclusion criteria were: pregnancy, significant heart disease, coagulopathy, allergy to bleomycin, lung and kidney dysfunction, implanted defibrillator or pacemaker, stage IV disease.

Table 1 summarizes the characteristics of patients affected by locally advanced pancreatic cancer and treated with electrochemotherapy.

### 2.2.Treatment Protocol

Electrochemotherapy was delivered through open laparotomy after a midline incision to allow staging of the disease and mobilization of the pancreatic tumor as reported in our previous publication [22].

**Figure 1:**
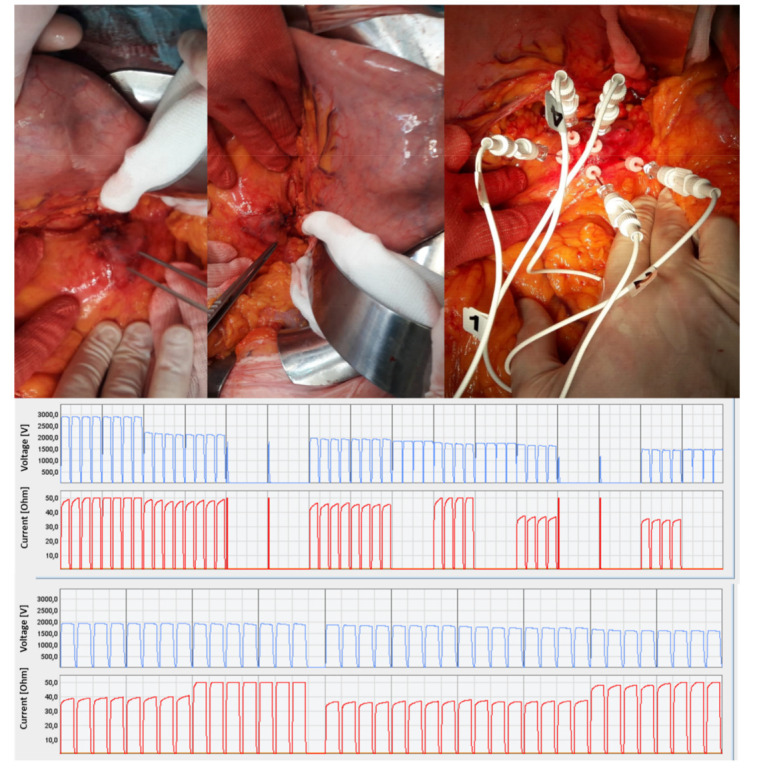
The panels show representative images of the open surgery procedure with electrode insertion in a variable geometry and the delivered electric pulses graphs.

**Figure 2:**
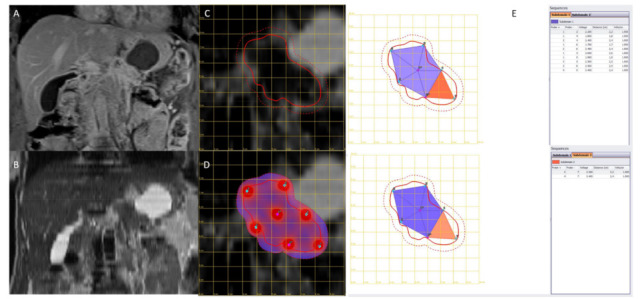
The panel reports an example of preoperative planning performed with the software Pulsar. In (**A**) (VIBE T1- weighted image post-contrast medium in coronal plane) and (**B**) (HASTE T2-weighted image in coronal plane) there are the magnetic resonance (MR) sequences used to identify the target areas, (**C**) represents the definition of the lesion contours, (**D**) represents the results of the planning in terms of number and position of the electrodes and (**E**) shows defined electrochemotherapy (ECT) treatment parameters for each subdomain (the treatment was divided into two subdomains, considering that the proposed optimized electrode number to obtain complete coverage of the lesion was 7).

Bleomycin was administrated intravenously (15,000 IU/m^2^) according to ESOPE (European Standard Operating Procedures of Electrochemotherapy) protocol [25,26]. Based on the localization and size of the pancreatic cancer, the surgeon decided to use needle electrodes with a linear fixed configuration (N-30-4B of IGEA S.p.A., Carpi, Italy), a hexagonal fixed configuration (N-30-HG or I-40-HG or H-30-ST of IGEA S.p.A., Carpi, Italy) or variable geometry (VGD1230 or VGD1240 of IGEA S.p.A., Carpi, Italy) using multiple insertions of a single needle (Figure 1). Treatment was completed within a window from 8 to 40 min after the end of the bleomycin bolus according to updated standardized operating procedures [26]. As reported in our previous publication [22]: “N-30-4B are needle electrodes hosting 8 needles, distributed on two rows of 4 needles each, 0.7 mm thick; needle electrodes have a gap of 4 mm between them; electrode needles are 30 mm long. N-30-HG and, I-40-HG and H-30-ST are needle electrodes hosting 7 needles. Six needles are at the vertices of a hexagon and the seventh is at its center with diameter size of 0.7 mm; electrode needles are 30 mm (N-30-HG and H-30-ST) or 40 mm long (I-40-HG). The latter with needles isolated for the first 2 cm (sheathed in polyethylene terephthalate) and active part of remaining 2 cm. H-30-ST are needle electrodes with active part adjustable from 5 to 30 mm to simplify the insertion in deeper-seated tumor nodule”. VGD1230 and VGD1240 electrodes are single-needle with a diameter of 1.2 mm, a length ranging from 16 to 24 cm and an active length, respectively, of 30 mm and 40 mm, and are mainly suitable for soft, deep-seated tumors; these electrodes are arranged in a geometry with variable configurations with a minimum of two needles and a maximum of six needles. A preoperative plan using the PULSAR software (IGEA S.p.A., Carpi, Italy) was performed when multiple insertion of single needles in a variable geometry was used. The use of the software allows estimation of the electric field required in the region of interest, calculating an optimized treatment in terms of electrode configuration (number and position), voltage and distance for each couple of electrodes within or around the predefined area segmented by the user (Figure 2) [27].

Electric pulses were delivered using the Cliniporator™ (IGEA S.p.A., Italy) with the following parameters: 8–96 pulses at 400–1000V (910–1000 V/cm) of 100 μs duration at 1-5000 Hz of repetition frequency or a single pulse for a single detected R-wave (ECG synchronization). Electric impulses were synchronized with the ECG with Accusync 42 medical device (IGEA S.p.A., Carpi, Italy).

Table 2 reports the electrode configuration for each ECT session.

### 2.3.Imaging Techniques

As established in the protocol, the patients underwent magnetic resonance imaging (MRI) and/or computed tomography (CT) and fluorodeoxyglucose position emission tomography (18F-FDG-PET) scans to baseline (before ECT) and post-ECT treatment at 1, 3, 6 and 12 months. The assessment at 1 month was for early response evaluation. Long-term follow-up was carried out with radiological imaging obtained every three months in the time thereafter. In this study, we considered the radiological findings at 1 and 6 months by ECT.

Magnetic Resonance (MR) and CT Protocol: 1.5T MR scanner (Magnetom Symphony, Siemens Medical System, Erlangen, Germany) equipped with a phased-array body coil was used for MRI acquisition. Morphological and functional sequences were performed, and details were reported in our previous study [28].

Sixty-four-slice MDCT scanner (Optima 660, GE Healthcare, Chicago, IL, USA) was used for non-contrast-enhanced phase and triple-phase contrast-enhanced CT scans; details were reported in Granata et al. manuscript [28].

Magnetic Resonance and CT Image Analysis: The images acquired before and after ECT were randomly and independently reviewed by four blinded observers with at least 10 years’ experience in MR and CT image interpretation of the pancreas. The response to ECT was evaluated according the RECIST 1.1 criteria [28,29,30]. For CT images, the response to ECT was evaluated according to the Choi criteria [31]. For functional MR analysis, we refer to our previously published article [28].

18F-fluorodeoxyglucose Positron Emission Tomography (18F-FDG PET) Data Acquisition and Images Analysis: 18F-FDG PET/CT studies were acquired 60 min after the administration of 300–385 MBq of FDG either with a Siemens ECAT EXACT 47 or a General Electric DST 600 PET-CT scanner. All calibrations on the scanners were regularly performed to obtain accurate FDG Standardized Uptake Value (SUV) readings. Patients fasted for at least 6 h, and blood glucose level was <150 mg/dL. Each patient underwent the baseline and the pre-operative study on the same scanner. Irregular volumes of interest (VOIs) were semi-automatically drawn by the expert investigator on orthogonal planes using a dedicated workstation and software using an arbitrary threshold, as reported previously [32,33]. For each patient, both studies were analyzed at the same time in order to minimize discrepancies in VOI positioning. For each study, maximum standardized uptake values (SUVmax) of the pancreas lesion were recorded. The analysis of 18F-FDG PET/CT results was performed by comparing measurements obtained in the pancreatic lesion at baseline (SUV1) and after treatment (SUV2). This change was expressed as the percentage of SUV reduction (ΔSUV = (SUV1−SUV2)/SUV1 × 100). Objective therapeutic responses were defined according to PERCIST 1.0 [32,33].

Local disease control rate (LDCR) represents the percentage of patients who have achieved complete response, partial response and stable disease.

### 2.4 Statistical Analysis

Data were expressed in terms of median value and range. Pain was assessed using the Defense and Veterans Pain Rating Scale (DVPRS) [34] with 10 levels (0 = No pain; 1 = Hardly noticeable pain; 2 = Noticeable pain, but does not interfere with activities; 3 = Somewhat distracting pain; 4 = Distracting pain, but does not affect normal activities; 5 = Pain interrupts some activities; 6 = Hard to ignore pain, avoidance of daily activities; 7 = Pain is the main focus of attention, prevents daily activities; 8 = Awful pain, difficult to do anything; 9 = Unbearable pain, cannot do anything; 10 = As bad as pain can be, nothing else matters). Pain evaluation was performed pre-ECT, after 1 month and after 6 months of ECT. Percentage of LDCR was reported. A chi-squared test was used to verify statistically significant differences in percentage values. A Mann–Whitney U test and Kruskal– Wallis test were performed to verify statistically significant differences between independent groups. Overall survival from ECT date estimates were calculated with Kaplan–Meier analysis.

A *p* value < 0.05 was considered statistically significant.

All analyses were performed using Statistics Toolbox of Matlab R2007a (The Math-Works Inc., Natick, MA, USA).

## 3. Results

### 3.1. Primary Endpoint Outcome (Local Disease Control and Overall Survival)

The median time between basal imaging assessment and ECT (range 7–14) was 9 days. The median time between ECT and first follow-up radiological assessment was 36 days (range 31–43), while between ECT and second radiological follow-up, assessment was 189 days (range 154–209). Twenty-five patients with a histological diagnosis of pancreatic adenocarcinoma were treated with ECT: 14/25 (56%) patients with tumors in the head of the pancreas and 11/25 (44%) patients with body/neck pancreatic tumors (Table 1). Needle electrodes with linear configurations were used in 3/25 (12%) patients, while electrodes with hexagonal configurations in 10/25 (40%) patients. Twelve patients (48%) were treated with multiple insertions of a single needle in a variable geometry configuration (Table 2).

**Figure 3:**
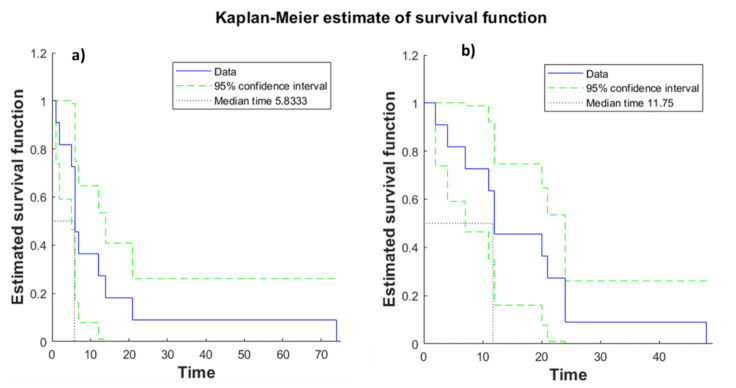
Overall survival curve (months) by Kaplan–Meier analysis in the two groups treated, respectively, with fixed (**a**) and variable geometry (**b**).

**Figure 4:**
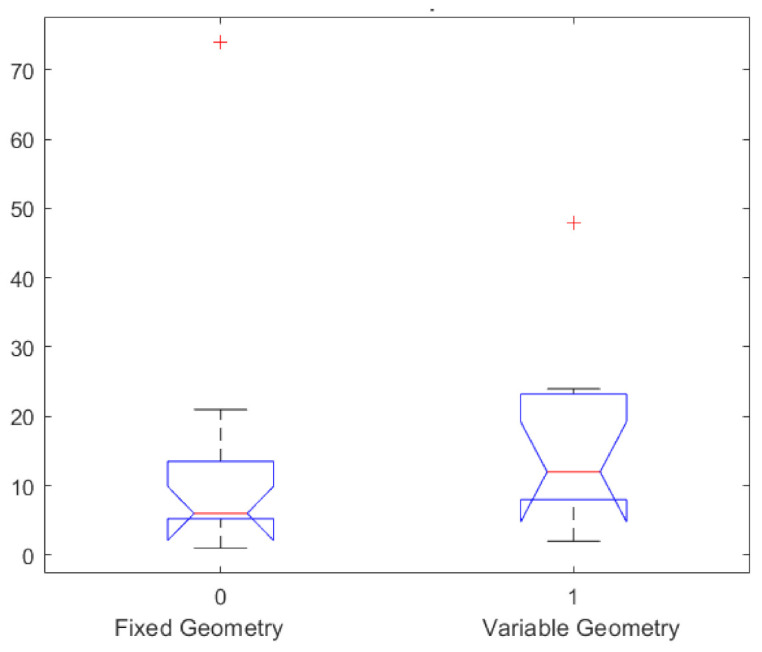
Boxplot of overall survival values in months in the two groups treated, respectively, with fixed and variable geometry.

Overall survival (OS) results by Kaplan–Meier analysis in terms of median value was 11.5 months (range 1–74 months). In the group of patients treated with fixed geometry OS, the median value (range values 1–74 months) was 6 months, while in the group of patients treated with variable geometry, the OS median value was 12 months (range 2–50 months, Table 2 and Figure 3). Therefore, there was a difference between the group treated with fixed geometry and the group treated with variable geometry with an improvement of overall survival of 4.5 months in the variable geometry group. However, this difference was not statistically significant using the Mann–Whitney U test (*p* value = 0.18). Figure 4 shows the boxplot of overall survival values between the two groups.

According to the abovementioned criteria for treatment response assessment, 1 month after ECT, 19/25 (76.0%) patients were in PR and 5/25 (20%) patients were in SD, while for 1 patient, the imaging was not available because of early exitus before the first follow-up (Table 3). Therefore, a LDCR of 96% (24/25) was reached one month after ECT.

Six months after the ECT, 11/25 (44%) patients were still in PR and 3/25 (12%) resulted in SD, while for 11/25 (44%) patients the imaging was not available because of early exitus before the second follow-up (Table 3). Therefore, a LDCR of 56% (14/25) was reached six months after ECT. The group of patients treated with fixed geometry had a LDCR of 46% (6/13), while in the group treated with variable geometry, the LDCR was 67% (8/12) (*p* value > 0.05).

**Figure 5:**
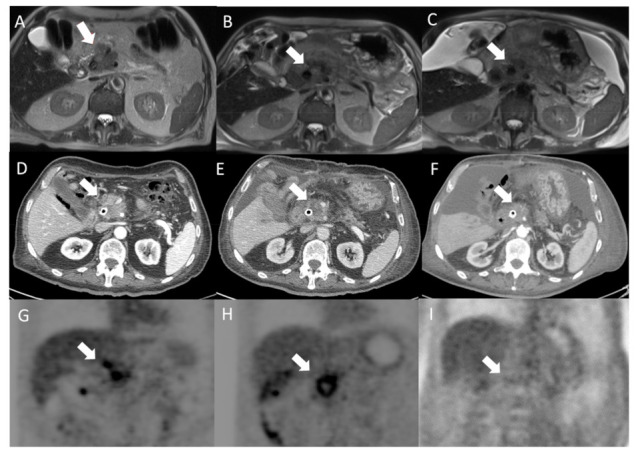
Head pancreatic cancer patient treated with fixed geometry. Before treatment, the arrow identifies the lesion on the HASTE T2-W sequence (**A**); after treatment the lesion (arrow) shows non-significant differences in signal intensity and size in (**B)** (one month after treatment) and in (**C**) (six months after treatment). In addition, non-significant difference on the CT study between pre- (**D**) and post-treatment was observed both after one month (**E**) and after six months by ECT (**F**). Instead, PET evaluation between pre- (**G**) and post-treatment detected a reduction of glucose uptake both after 1 month (**H**) and after 6 months (**I**) by ECT. According to data, the patient, after one month and after six months, was classified to be in stable disease by ECT.

Figure 5 reports representative images before and after treatment of a patient treated with fixed geometry. After treatment, on MR images, there were not significant differences in signal intensity and lesion size; after six months from ECT, the HASTE T2 weighted (T2-W) sequence shows again a stable disease in terms both of signal intensity and lesion size. On CT, both after one month and after six months from ECT, there was not a significant reduction of the density and lesion size. Instead, 18F-FDG PET/CT evaluation detected a reduction of glucose uptake both after 1 month and after 6 months of ECT. However, according to data, the patient, after one month and after six months from ECT, was classified as having stable disease.

**Figure 6:**
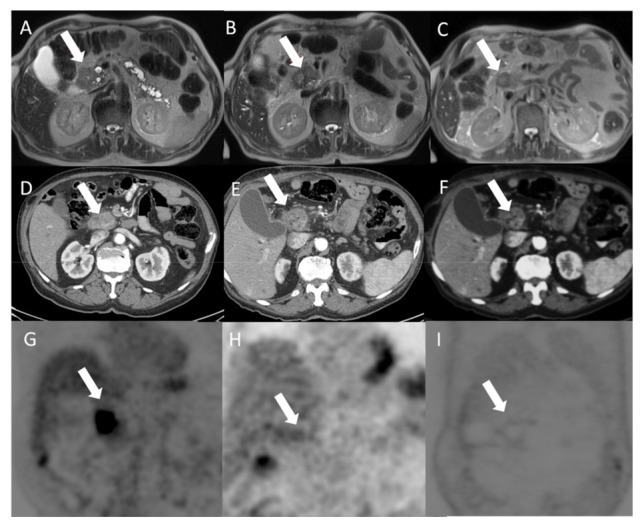
Patient with adenocarcinoma of the pancreatic head treated with variable geometry. Before treatment, the arrow identifies the lesion on the HASTE T2-W sequence (**A**); after one month of treatment, on the HASTE T2-W sequence (**B**), there were not significant differences in signal intensity and lesion size; after six months from ECT, the HASTE T2-W sequence (**C**) showed significant differences in signal intensity and lesion size. In the pancreatic phase of the CT study (**D**), the lesion appeared hypodense (arrow). After one month of treatment, the lesion appeared more hypodense (**E**); after six months of treatment, on CT (**F**), there was also a significant reduction of the lesion size. Also, PET evaluation between pre- (**G**) and post-treatment detected a reduction of glucose uptake both after 1 month (**H**) and after 6 months (**I**) by ECT. According to data, the patient, after one month and after six months, was classified to be in partial response by ECT.

Figure 6 reports representative images before and after treatment of a patient treated with variable geometry. After one month, on MR images, there were not significant differences in signal intensity and lesion size, while, after six months from ECT, the HASTE T2-W sequence showed significant differences in signal intensity and lesion size. On the CT study, after one month, the lesion appeared more hypodense, and after six months, there was a significant reduction of the lesion size. In addition, 18F-FDG PET/CT evaluation between pre- and post-treatment detected a reduction of glucose uptake both after 1 month and after 6 months from ECT. According to data, the patient, after one month and after six months from ECT, was classified as a partial response.

### 3.2. Secondary Endpoints Assessment (Feasibility and Safety)

**Figure 7:**
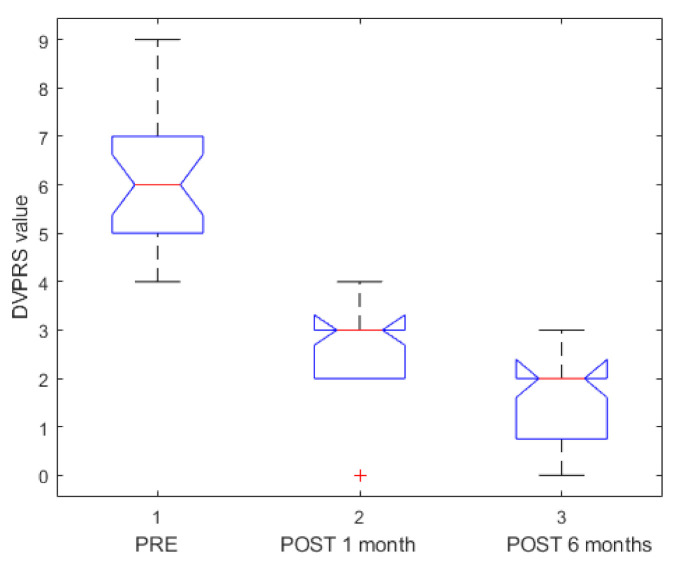
Boxplot of Defense and Veterans Pain Rating Scale (DVPRS) assessed pre-, after 1 month and after 6 months of ECT.

As shown in Table 2, ECT was well-tolerated, and abdominal pain was rapidly resolved (4–8 days; median range 6.5 days). Figure 7 shows a boxplot of DVPRS pre-ECT, 1 month after and 6 months after ECT (*p* value < 0.01 at Kruskal–Wallis test). Serious adverse events that were electrochemotherapy-related had not occurred. Heart abnormalities were not reported during EP pulse delivery. Only one patient in the series had a transient, self-limiting, supraventricular arrhythmia. No significant arrhythmias or myocardial ischemia after ECT were detected.

During or following ECT, clinically significant hemodynamic or serum biologic changes were not observed. A slight increase in lipase levels of all patients was registered, but they returned to normal within 72 h, while no significant elevations in serum amylase were present. No one had bleeding or damage surrounding viscera or vascular structures.

3/25 (12%) patients, with biliary stents, had venous stasis of the duodenum reported by CT, one month after ECT.

13/25 (52%) patients reported hyperpyrexia with rapid resolution in 1–3 days. 7/25 (28%) patients, one month after ECT, saw delayed gastric emptying without clinically significant signs, as reported by CT and MR. The delayed gastric emptying did not require treatment.

Ascites was observed in 8/25 (32%) patients after treatment, while pleural effusion was present in 6/25 (24%) patients. Both ascites and pleural effusion were probably due to the pancreatic inflammation induced by the treatment, did not require treatment and resolved spontaneously.

Computed tomography and MR images at one month after ECT showed splenic infarction without thrombosis of the splenic vessels (Table 4, which did not require treatment. The median duration of hospitalization was 11.7 days (range 7–19).

## 4. Discussion

Adenocarcinoma of the pancreas is the one of the most common and aggressive forms of cancer, and surgical resection is often the unique, potentially curative treatment option. When possible, resection plus chemotherapy is the treatment of choice in order to increase survival. The most common complications after pancreatoduodenectomy are pancreatic fistula, which represent the major source of morbidity [35,36]. About 40% of the patients died of septic and hemorrhagic complications following pancreatic fistula, and the current derivative surgical techniques (pancreatico-jejunostomy, pancreatico-gastrostomy and duct occlusion) proposed to manage the pancreatic stump are still debated [35,36]. Recent therapeutic approaches are aimed to obtained tumor debulking or interstitial ablation after the failure of first-line treatments in locally advanced and metastatic pancreatic cancer [5,6,7,8] because current standard therapies such as radiotherapy and chemotherapy alone or in combination are efficacious only in a limited group of patients. In addition, radiotherapy combined with chemotherapy can be considered to treat locally advanced, unresectable pancreatic cancer with methods such as stereotactic body radiotherapy [37,38,39]. In our population, the patients had not undergone radiotherapy; however, an interesting future study could be to assess electrochemotherapy versus radiotherapy in locally advanced patients not suitable for surgery without progressive disease.

Some initial clinical studies of different thermal ablation techniques were associated with significant morbidity and mortality as a result of duodenum or peripancreatic vessel damage [10,11]. For these reasons, their adoption was limited and non-thermal alternative ablative approaches for treatment of LAPC were followed. Techniques in which short, high-voltage pulses are applied to tissues to permeabilize the cell membranes reversibly and temporarily (ECT) [14,15,16] or irreversibly, causing cell death (irreversible electroporation, IRE) [38,39,40,41,42] have been tested in clinical trials. The cell membrane can be permeabilized, though the optimal mechanism through which electrical pulses permeabilize the cell membrane is not completely understood from a frequency or repetition standpoint, with outcomes depending on pulse amplitude, duration and the number of pulses [14,15,40].

Electrochemotherapy and IRE can be both safely used to treat LAPC patients. Vital structures such as larger blood vessels, nerves or viscera are not damaged by ECT [14,15], and IRE does not involve significant risk of pancreatitis or surrounding vascular injury-thrombosis [43,44,45]. Irreversible electroporation combined with chemotherapy represents a new treatment modality of patients with LAPC and shows an improved efficacy in terms of cancer-specific survival (CSS) and progression-free survival (PFS) rates compared with chemotherapy alone [46]. Many studies seem to confirm that induction therapy followed by IRE can improve survival of LAPC patients. A total of 132 patients with LAPC underwent induction chemotherapy followed by IRE, and an improvement of OS and PFS were obtained in comparison to chemotherapy alone [47]. This combined treatment for LAPC is a safe treatment and as suggested by Yang “for well-selected patients, IRE can achieve encouraging survival outcomes” [48]. This result was confirmed by Martin et al. in a study on 200 patients with LAPC treated with IRE alone (*n* = 150) or pancreatic resection plus IRE for margin enhancement (*n* = 50). Addition of IRE to induction chemotherapy and chemoradiation therapy results in substantially prolonged survival compared with historical controls [49].

Holland et al. [50] treated 152 patients with IRE in LAPC with open technique following the American Hepato-Pancreato-Biliary Association (AHPBA) Registry Protocols. All patients underwent successful IRE. Morbidity and mortality were 18% and 2%, respectively, with 19 (13%) patients experiencing severe adverse events. Nine (6%) patients presented with local recurrence. Median PFS and OS from diagnosis were 22.8 months and 30.7 months respectively.

The combination of IRE with established multi-agent therapy is safe and demonstrates encouraging survival among patients with LAPC. IRE is associated with a low rate of serious adverse events and has been optimized for more widespread adoption through the standardized protocols available through the AHPBA registry [50].

In this study, we used reversible electroporation. The feasibility and effectiveness of electrochemotherapy on deep tumors was already shown [18,23,24,25,51], and the safety and feasibility of ECT on pancreatic tumors was reported in our previous work [18]. No side effects nor major complications, no clinically relevant elevation of amylase and lipase levels nor evidence of clinical pancreatitis were observed in the treated patients.

Electrochemotherapy is definitely a promising technique for cancer treatment, [16,22,24] but how to assess treated tumor response is still the problem [52,53,54,55,56,57,58,59,60,61,62,63,64]. In fact, as highlighted in our preliminary experience, the RECIST 1.1, using the variation of largest diameter both on CT and MR images, did not provide an appropriate patient stratification in responders or non-responders after ECT [22]. The RECIST criteria restrictions in the assessment of residual viable tumors of treated hepatocellular carcinoma (HCC) and in gastrointestinal stromal tumors (GIST) are well known [32]. Electrochemotherapy potentiates the cytotoxic effect of chemotherapy and therefore, the Choi criteria would appear to be more suitable for early treatment evaluation [34,49].

In our cohort, overall survival was 11.5 months in terms of median value. Interestingly, in the group of 13 patients treated with fixed geometry electrodes, OS was 6 months, while in the group of 12 patients treated with needles in variable geometry, the OS median value was 12 months. Therefore, an improvement of overall survival of 4.5 months in the group of patients treated with customized treatment was observed. Electrochemotherapy also looks promising in terms of LDCR (percentage of patients who have achieved complete and partial response and stable disease). One month after treatment, a LDCR of 96.0% was reached, and six months after the ECT, a LDCR of 56.0% was reached. A local disease control rate of 46% was observed in the group of patients treated with fixed geometry versus 67% observed in the group treated with variable geometry, confirming an advantage with this kind of electrode configuration.

Even if these differences were not statistically different, it is suggestive that a personalized treatment plan ensuring a more complete coverage of the tumoral lesion may determine an improvement in local disease control and in overall survival as a consequence of a more efficient and complete treatment. We believe that a study with a larger number of patients will help confirm this evidence.

Our results confirmed that electrochemotherapy on LAPC can be performed safely and feasibly. In fact, a good functional result was obtained without recording of side effects or major complications. No evidence of clinical pancreatitis was present, although in 8 patients, ascites was seen, and in 6 patients, pleural effusion, explainable as a result of pancreatic inflammation induced by the treatment. There was no evidence of intraoperative bleeding, no evidence of pancreatic fistula or damage to surrounding viscera except venous stasis of the duodenum in three patients with biliary stents and delayed gastric emptying in 7 patients without clinically significant signs. Moreover, the patients reported pain reduction after 1 month and after 6 months from the treatment compared to preoperative status as well as better quality of life.

None of our patients showed signs of thermal damage after ECT treatment. Thermal damage has been described due to the high currents that are delivered by ECT [48]. In our previous study, we observed that this is a main concern with needle electrodes that have a very small diameter, and this shape can result in a very high local current density. Hexagonal configuration has a better chance for the lesions falling at the center of the electromagnetic field without a need for a very high local current density [22].

In addition to the sample size, a limitation of the study is the non-randomized nature of the trial with the absence of a control group. A randomized trial, with the aim to evaluate the efficacy of electrochemotherapy followed by conventional systemic treatment as compared to only systemic treatment in LAPC in terms of objective response, is going to start in our institute to fill this gap.

The use of ECT and IRE in deep cancer, e.g., liver and pancreas, currently requires a laparotomy surgical approach and limits its applicability due to the risks associated with open surgery. Hopefully, in the future, thanks to the technical advances of a new generation of electrodes, it might be possible to perform ECT laparoscopically with a minimally invasive approach instead of standard open laparotomy.

## 5. Conclusions

Electrochemotherapy on LAPC can be performed safely and feasibly. A good functional result was obtained without recording of side effects or major complications.

Electrochemotherapy obtained good results in terms of the local disease control rate in LAPC and was potentially able to improve overall survival.

A lower percentage of local disease control rate was observed in the group of patients treated with fixed geometry versus the group treated with variable geometry, suggesting an advantage with this kind of electrode configuration. Even if these differences were not statistically different, it suggests that personalized treatment planning, with multiple insertions with variable geometry ensuring a more complete coverage of the tumoral lesion, may determine both an improvement in local disease control and in overall survival.

## Figures and Tables

**Table 1:** Characteristics of locally advanced pancreatic cancer patients treated with electrochemotherapy.

Patients (*n* = 25)	
Histotype %	
Adenocarcinoma	100 (25/25)
Location %	
Head	56.0 (14/25)
Body/tail	44.0 (11/25)
Venus involvement (SMV or PV) %	
Yes	84.0 (21/25)
No	16.0 (4/25)
Arterial encasement %	
Yes	56.0 (14/25)
No	44.0 (11/25)

**Table 2:** Electrode configuration for each treatment and overall survival results in terms of median and range values.

Patient			Electrodes Configuration		OS [Months] by Kaplan Meier Analysis		Pain Assessed by DVPRS		
No.	Age	Sex	Geometry	Model			PRE ECT	POST 1 Month	POST 6 Months
Median Value (Range) = 68 (34)			13 Treatments with Fixed Geometry and 12 Treatments with Variable Geometry		Median Value (Range) = 11.5 (1–74)		Median Value Pre (Range) = 6 (4–9)	Median Value Pre (Range) = 3 (2–4)	Median Value Pre (Range) = 2 (1–3)
1	48	M	Hexagonal	N-30-HG	median value (range)	6 (1–74)	6 (4–8)	2.5 (2–4)	2 (1–3)
2	63	F	Hexagonal	I-40-HG					
3	71	F	Hexagonal	N-30-HG					
4	61	F	Linear	N-30-4B					
5	72	F	Hexagonal	N-30-4B					
6	80	F	Hexagonal	N-30-HG					
7	60	F	Linear	N-30-4B					
8	62	F	Linear	N-30-4B					
9	67	M	Hexagonal	N-30-HG					
10	57	M	Hexagonal	I-40-HG					
11	74	M	Hexagonal	H-30-ST					
12	67	M	Hexagonal	I-40-HG					
13	68	M	Hexagonal	I-40-HG					
14	79	M	Multiple single-needle in a variable geometry	VGD1240	median value (range)	12 (2–48)	6.5 (4–9)	3 (2–4)	2 (1–3)
15	71	M	Multiple single-needle in a variable geometry	VGD1240					
16	80	M	Multiple single-needle in a variable geometry	VGD1240					
17	82	M	Multiple single-needle in a variable geometry	VGD1230					
18	62	F	Multiple single-needle in a variable geometry	VGD1230					
19	63	F	Multiple single-needle in a variable geometry	VGD1230					
20	64	F	Multiple single-needle in a variable geometry	VGD1240					
21	68	F	Multiple single-needle in a variable geometry	VGD1230					
22	70	F	Multiple single-needle in a variable geometry	VGD1230					
23	81	M	Multiple single-needle in a variable geometry	VGD1230					
24	79	F	Multiple single-needle in a variable geometry	VGD1230					
25	64	F	Multiple single-needle in a variable geometry	VGD1230					

Note. OS: overall survival; DVPRS: Defense and Veterans Pain Rating Scale; PRE ECT: Before Electrochemotherapy

**Table 3:** Tumor size before and after electrochemotherapy for individual patients evaluated by magnetic resonance and computed tomography.

Patient	Tumor size		Tumor Response After 1 Month by ECT Treatment							Tumor Response After 6 Months by ECT Treatment						
No.	CT (mm)	MR (mm)	1st Radiological Evaluation after ECT (CT); Size (mm)	ΔCT Largest Diameter (%)	ΔHU (%)	1st Radiological Evaluation After ECT (MR); Size (mm)	ΔMR Largest Diameter (%)	ΔSUVmax (%)	Response Assessment According to Granata et al. [27]	2nd Radiological Evaluation After ECT (CT); size (mm)	ΔCT Largest Diameter (%)	ΔHU (%)	2nd Radiological Evaluation After ECT (MR); size (mm)	ΔMR Largest Diameter (%)	ΔSUVmax (%)	Response Assessment According to Granata et al. [27]
1	99	95	90	11.6	22.7	87	8.4	−177.8	PR	-	-	-	-	-	-	-
2	43	48	38	9.1	40.4	43	10.4	-	PR	-	-	-	-	-	-	-
3	59	64	54	8.5	34	57	11.5	38.5	PR	28	52.5	54.6	27	57.7	-	PR
4	22	26	19	13.6	7.8	23	2	-	PR	42	−90.9	25.9	-	-	-	PR
5	51	49	49	3.9	48.7	-	-	-	PR	50	2	43.6	-	-	-	PR
6	48	-	45	6.3	18.9	-	-	-	PR	-	-	-	-	-	-	-
7	33	-	24	27.3	49.5	-	-	-	PR	-	-	-	-	-	-	-
8	30	-	22	26.7	51.6	-	-	-	PR	-	-	-	-	-	-	-
9	99	-	-	-	-	-	-	-	-	-	-	-	-	-	-	-
10	56	-	46	17.9	42.6	-	-	100.0	PR	-	-	-	-	-	-	-
11	56	58	59	−5.4	49.1	51	12.1	66.5	SD	36	35.7	43.2	37	36.2	84.0	PR
12	63	68	55	12.7	6.8	55	19.1	−17.9	SD	57	9.5	12.4	50	26.5	18.9	SD
13	28	30	28	6.7	44.4	24	20	46.8	PR	35	-25	36.9	-	-	21.3	PR
14	50	41	46	8	44.8	38	7.3	44.8	PR	58	−16	76.3	36	12.2	100.0	PR
15	35	34	56	−60	83.3	-	-	-	PR	-	-	-	-	-	-	-
16	53	-	49	7.5	23.4	-	-	-	PR	-	-	-	-	-	-	-
17	64	55	49	23.4	35.5	46	16.4	18.8	PR	60	6.3	23.9	42	23.6	-	PR
18	51	51	66	−29.4	40	65	−9.8	17.0	PR	-	-	-	-	-	-	-
19	53	53	50	5.7	44	49	7.5	32.3	PR	36	32.1	45.8	-	-	55.0	PR
20	54	50	53	1.9	13.3	50	0	27.4	SD	-	-	-	-	-	-	-
21	-	35	-	-	-	35	0	−13.6	SD	-	-	-	20	42.9	43.2	PR
22	40	41	43	−7.5	46.6	42	−2.4	-	PR	10	75	48.9	-	-	-	PR
23	40	-	37	7.5	35.8	-	-	−12.1	PR	55	−37.5	14.5	-	-	-	SD
24	36	20	36	0	54.1	35	−75.0	47.0	PR	25	30.6	32.7	20	0	-	PR
25	52	65	54	−3.8	12.4	62	4.6	2.7	SD	60	−15.4	12.9	60	7.7	-	SD

Note. ECT: electrochemotherapy; CT: computed tomography; MR: magnetic resonance; ΔCT: variation of the largest diameter on CT between pre- and post-treatment; ΔMR: variation of the largest diameter on CT between pre-and post-treatment; ΔSUVmax: variation of the SUVmax between pre- and post-treatment; PR = partial response; SD = stable disease

**Table 4:** Feasibility and Safety results.

Complications after Treatment	13 Treatments with Fixed Geometry	12 Treatments with Variable Geometry
hyperpyrexia	7/13 (53.8%)	6/12 (50.0%)
Delayed gastric emptying without clinically significant signs	4/13 (30.8%)	3/12 (25.0%)
ascites	6/13 (46.1%)	2/12 (16.7%)
splenic infarction without thrombosis of the splenic vessels	3/13 (23.1%)	0/12 (0%)
pleural effusion	4/13 (30.8%)	2 (16.7%)
